# Bipolar disorder and risk of all-cause and specific dementia: a systematic review and meta-analysis

**DOI:** 10.3389/fnagi.2026.1980508

**Published:** 2026-10-13

**Authors:** Zihan Gao, Meiling Ren, Ruiji Yang, Jiahui Yu

**Affiliations:** School of Public Health, Shanxi Medical University, Taiyuan, China

**Keywords:** Alzheimer’s disease, bipolar disorder, dementia, meta-analysis, risk, systematic review, vascular dementia

## Abstract

**Background:**

Bipolar disorder (BD) is associated with cognitive impairment and an increased risk of dementia; however, the magnitude of this association and subtype-specific risk patterns remain unclear. This systematic review and meta-analysis quantified the associations between BD and the risks of all-cause dementia, Alzheimer’s disease (AD), and vascular dementia (VaD), and explored potential sources of between-study heterogeneity.

**Methods:**

PubMed, Embase, the Cochrane Library, and Web of Science were searched from inception to September 15, 2026. Eligible cohort and case–control studies reported adjusted effect estimates for the association between BD and dementia. Hazard ratios and odds ratios were converted to risk ratios (RRs) prior to quantitative synthesis. Random- or fixed-effects models were applied according to heterogeneity. Leave-one-out sensitivity analyses, Egger’s test for publication bias, and subgroup analyses stratified by follow-up duration, sex, age at BD onset, and study design were conducted.

**Results:**

Eleven studies involving 1,536,508 participants and 90,864 all-cause dementia cases were included. BD was associated with an increased risk of all-cause dementia (RR = 3.55, 95% CI 2.48–5.07; I^2^ = 98.7%), AD (RR = 5.08, 95% CI 1.87–13.84; I^2^ = 97.2%), and VaD (RR = 4.33, 95% CI 3.06–6.13; I^2^ = 71.7%). Estimates for all-cause dementia and VaD were robust in sensitivity analyses, whereas the AD estimate was sensitive to the exclusion of individual studies and should therefore be interpreted cautiously. The association between BD and increased dementia risk was observed across the examined subgroups. Formal tests for subgroup differences showed no statistically significant differences according to sex or age at BD onset.

**Conclusion:**

BD is associated with an increased risk of all-cause dementia, AD, and VaD. However, substantial between-study heterogeneity and the limited evidence available for specific dementia subtypes warrant cautious interpretation of the pooled estimates. Further high-quality longitudinal studies are needed to clarify the factors underlying heterogeneity and subtype-specific associations.

**Systematic review registration:**

https://www.crd.york.ac.uk/prospero/, identifier [CRD420261395626].

## 1 Introduction

Bipolar disorder (BD) is a chronic and recurrent mood disorder characterized by episodes of mania or hypomania and depression. It has an estimated global prevalence of 0.5% (World Health Organization, 2025), with onset typically occurring during adolescence or early adulthood. Beyond its considerable impact on psychosocial functioning and quality of life, BD is often accompanied by persistent impairments in attention, memory, and executive function that can persist even during euthymic periods (Swidzinski et al., 2025). As BD usually emerges in adolescence or early adulthood and follows a lifelong relapsing trajectory, affected individuals endure prolonged exposure to illness-related, behavioural, and treatment-related factors that can negatively influence cognitive health. BD is therefore a major cause of disability and poses an important long-term public health challenge.

Dementia is defined by progressive cognitive decline severe enough to compromise independent functioning. Its prevalence rises alongside population ageing, imposing substantial clinical, social, and economic burdens on patients, caregivers, and healthcare systems. Alzheimer’s disease (AD) and vascular dementia (VaD) represent the most prevalent dementia subtypes. AD is driven largely by progressive neurodegeneration, while VaD results from cerebrovascular injury. Given their distinct underlying mechanisms and risk profiles, the associations between BD and subtypes require separate evaluation. Cumulative clinical and epidemiological evidence indicates that people with BD experience faster cognitive decline and higher dementia incidence compared with the general population (Kessing and Munkholm, 2026). Even so, the magnitude of this association and its specificity across dementia subtypes remain unclear.

Existing cohort and case–control studies have produced conflicting effect estimates. Several studies have reported that BD is linked to a two-fold to seven-fold elevation in dementia risk, while other investigations, especially those with small sample sizes, have documented weak or statistically non-significant associations. Variation in geographical setting, follow-up duration, sex distribution, age at BD onset, and study design including prospective and retrospective approaches may explain such discrepancies (Kessing and Munkholm, 2026). Further limitations include wide variation in sample size, inconsistent reporting of dementia subtypes, heterogeneous adjustment for confounders such as age, vascular comorbidities, medication use and educational attainment, and limited data on illness duration (Diniz et al., 2017; Velosa et al., 2020). Most primary studies focus only on all-cause dementia, meaning risks specific to AD and VaD remain poorly quantified. In addition, prior evidence syntheses have rarely combined subtype-specific outcomes with comprehensive subgroup and sensitivity analyses, so the true relationships between BD and individual dementia subtypes are not fully established (Kessing and Munkholm, 2026). Against this background, we performed this meta-analysis to investigate the associations of bipolar disorder (BD) with risks of all-cause dementia and specific dementia subtypes and provide the best available evidence.

## 2 Method

This systematic review and meta-analysis adhered to the updated Preferred Reporting Items for Systematic Reviews and Meta-Analyses (PRISMA) statement (Page et al., 2020). The protocol for this study was prospectively registered with PROSPERO (CRD420261395626). According to the PICOS framework, the research question was defined as follows: Population (P), individuals diagnosed with bipolar disorder (BD); Intervention/Exposure (I), bipolar disorder; Comparator (C), individuals without bipolar disorder in cohort studies and individuals without dementia in case–control studies; Outcomes (O), all-cause dementia, Alzheimer’s disease (AD), and vascular dementia (VaD) with dementia diagnoses identified using the International Classification of Diseases, Ninth or Tenth Revision (ICD-9 or ICD-10) diagnostic codes; and Study design (S), cohort and case–control studies. For quantitative synthesis, the effect-measure framework used in this meta-analysis was based on adjusted hazard ratios (HRs) and odds ratios (ORs), together with their corresponding 95% confidence intervals (CIs).

### 2.1 Data sources and searches

A comprehensive literature search was performed across four databases (PubMed, Embase, Cochrane Library, and the Web Of Science) for cohort studies published from database inception through September 15, 2026. The search strategy incorporated a combination of Medical Subject Headings (MeSH) terms and free-text keywords, including “Bipolar Disorder,” “Dementia,” and “risk” (Supplementary Tables 1–4).

### 2.2 Study selection

Inclusion criteria: Exposure: a clear diagnostic definition of bipolar disorder; Control: for cohort studies, individuals without bipolar disorder, and for case–control studies, individuals without dementia, with bipolar disorder exposure ascertained comparably between cases and controls; Outcome: all-cause dementia or specific types of dementia, with dementia diagnoses identified using ICD-9 or ICD-10 diagnostic codes, with sufficient data to extract adjusted hazard ratios (HRs) or odds ratios (ORs) and their corresponding 95% confidence intervals (CIs); Study design: cohort studies or case–control studies. Conference abstracts, study protocols, duplicate publications, and studies that did not provide an eligible comparator or sufficient data for quantitative synthesis were excluded. When multiple reports were derived from the same underlying database or population, we assessed the degree of overlap based on study period, participant selection, exposure definition, and outcome ascertainment. Studies were not excluded solely because they used the same database; however, when substantial overlap in the underlying study population was likely and the same exposure–outcome association was evaluated, only the most recent or most directly relevant report was retained to avoid double-counting participants. Two reviewers (ZG and RY) independently assessed the eligibility of all retrieved records against the eligibility criteria. Disagreements were resolved by a third author (JY).

### 2.3 Data extraction

Relevant data were extracted by two independent authors (MR and ZG) and discrepancies were resolved by a third author (RY). The extracted information included the first author’s name, publication year, study location, sex, age, sample size, number of dementia cases, follow-up duration, bipolar disorder subtype, and specific incidence outcomes.

### 2.4 Quality assessment

The quality of the included studies was assessed using the Newcastle-Ottawa Scale (NOS) (Higgins et al., 2023), which assigns scores from 0 to 9, with score ≥7 indicating high quality; 4–6 moderate quality; and ≤3 low quality.

### 2.5 Statistical analysis

The adjusted effect estimates and 95% confidence intervals (CIs) from individual studies were used to calculate the effect sizes for bipolar disorder and the risks of all-cause and cause-specific dementia. To facilitate the quantitative synthesis of studies reporting different measures of association, the reported hazard ratios (HRs) and odds ratios (ORs) were converted to risk ratios (RRs) before meta-analysis using established statistical methods. Specifically, ORs were converted to approximate RRs based on the outcome risk in the reference group according to the method proposed by Zhang and Yu (1998). HRs were converted to approximate RRs using the method proposed by VanderWeele (2020), which provides an optimal approximate conversion under the proportional hazards assumption. These conversions were performed to place the effect estimates from different studies on a common RR scale and thereby enable their combined analysis. Heterogeneity between studies was evaluated using Cochran’s Q test and I^2^ statistics (Higgins et al., 2003). When the I^2^ value exceeded 50%, a random-effects model was adopted for meta-analysis; otherwise, a fixed-effects model was employed (DerSimonian and Laird, 1986). A sensitivity analysis was performed by sequentially excluding one study at a time to assess the robustness of the overall findings (Meng et al., 2024). In addition, to assess the potential influence of overlapping study populations among studies derived from the Taiwan National Health Insurance Research Database (NHIRD), we conducted additional sensitivity analyses in which only one of the potentially overlapping Taiwanese studies was retained at a time and the remaining Taiwanese studies were excluded from the corresponding analysis. Publication bias was assessed visually via funnel plots, and Egger’s regression test was conducted to statistically assess the bias (Egger et al., 1997). Subgroup analyses were performed according to follow-up duration, sex, onset age, and study design to detect potential sources of heterogeneity (Borenstein and Higgins, 2013). All statistical analyses were performed using Stata statistical software (Version 16.0; StataCorp, College Station, TX, USA).

## 3 Results

### 3.1 Study selection

A preliminary search across four major electronic databases yielded 3,281 records. After duplicates were removed, 2,493 records remained for title and abstract screening. Of these, 2,477 records were excluded, including 333 review articles and 2,144 articles that were not relevant to the research question. Following full-text review, five additional studies were excluded: three did not meet the comparator eligibility criteria, and two did not report relevant outcomes. Ultimately, 11 studies were included in the final meta-analysis (Figure 1).

**Figure 1:**
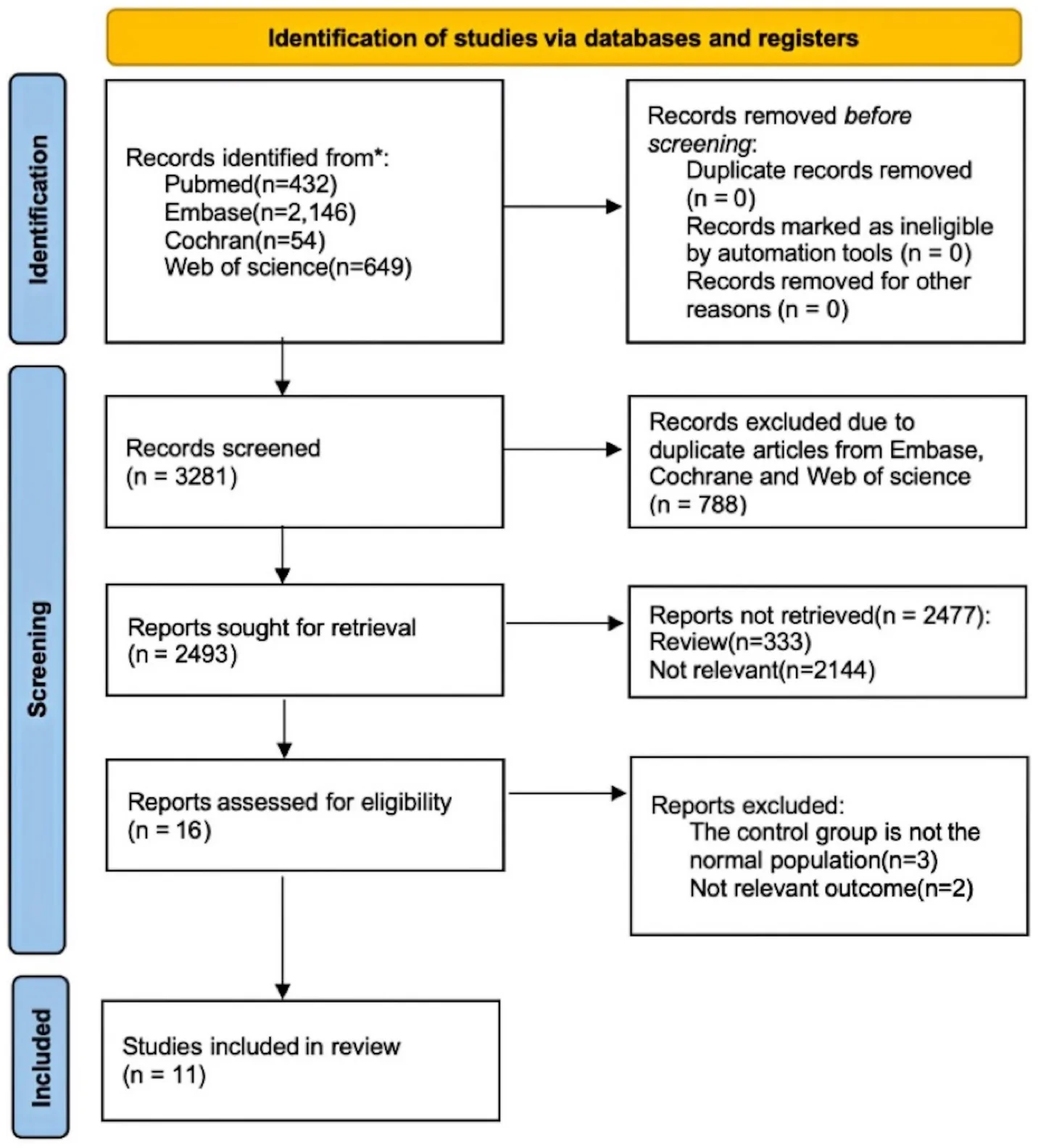
Study flowchart depicting the methodology used in selecting articles for inclusion in the study.

### 3.2 Study characteristics

Eleven studies (Ma'u et al., 2026; Nielsen et al., 2024; Xu et al., 2024; Liu et al., 2024; Liou et al., 2023; Chang et al., 2023; Kershenbaum et al., 2021; Almeida et al., 2016; Chen et al., 2015; Wu et al., 2013; Lin et al., 2020) met the final inclusion and exclusion criteria (Table 1). Follow-up durations across the included studies ranged from 5 to 14 years. The studies included a total of 1,536,508 participants, among whom 90,864 cases of all-cause dementia were reported. The included studies comprised two studies from Oceania, four from Europe, and five from Taiwan, China. Of these, four were prospective cohort studies, six were retrospective cohort studies, and one was a case–control study. All studies accounted for potential confounders using multivariable adjustment. Regarding dementia outcomes, 10 studies evaluated all-cause dementia, four evaluated Alzheimer’s disease, and three evaluated vascular dementia. Ten studies included participants of both sexes, whereas one included only men. The NOS scores of the individual studies ranged from 5 to 8 (Supplementary Table 5).

**Table 1:** Characteristics of the included studies.

Author	Year	Country	Study Type	Participants source	Follow-up period	Dementia diagnostic criteria	Case group	Control group	Age	Female	Type
Ma'u et al. (2026)	2025	New Zealand	Prospective Cohort study	New Zealand Health Survey (NZHS)	2011.12–2022.4	ICD-9/ICD-10	993	73,191	> = 30	58%	All-cause dementia
Nielsen et al. (2024)	2024	Denmark	Retrospective Cohort study	Danish Civil Registration System (CPR)	2007.8–2020.10	ICD-10	37,084	189,662	≥18 (Case group 46.1; Control group 46.6)	59.50%	All-cause dementia
Xu et al. (2024)	2024	UK	Prospective cohort study	UK Biobank	13.8 years	ICD-10/SCID-I	2,250	498,983	40–69 (average 56.5)	0.544	All-cause dementia, Alzheimer’s disease, vascular dementia
Liu et al. (2024)	2024	UK	Prospective cohort study	UK Biobank	2009.1–2024.1	ICD-10	1,076	446,733	40–69 (average 55.6)	Case group 56.0% (Control group 53.9%)	Alzheimer’s disease
Liou et al. (2023)	2023	Taiwan	Retrospective cohort study	National Health Insurance Research Database(NHIRD)	2006.12.31–2011.12.31	ICD-9-CM	14,138	42,412	45–69 (average 54.5)	Case group 42.1% (Control group 57.9%)	All-cause dementia, Alzheimer’s disease, vascular dementia, Non-special dementia
Chang et al. (2023)	2023	Taiwan	Retrospective cohort study	NHIRD	2008.1.1–2015.12.31	ICD-9-CM	4,883	108,084	≥60 (Case group 68.6; Control group 68.7)	Case group 56.7% (Control group 64%)	All-cause dementia
Kershenbaum et al. (2021)	2021	UK	Retrospective cohort study	CPFT Research Database	2013.1.1–2019.8.19	ICD-10					All-cause dementia
Almeida et al. (2016)	2016	Australia	Prospective cohort study	Health In Men Study (HIMS)	1996.4.2–2009.6.30	ICD-8/9/10	256	37,512	65–85 (average 72.5)	0%	All-cause dementia
Chen et al. (2015)	2015	Taiwan	Retrospective cohort study	NHIRD	1998.1.1–2011.12.31	ICD-9-CM	345	2,291	≥55 (average 64.72)	Case group 57.4% (Control group 64.2%)	All-cause dementia
Wu et al. (2013)	2013	Taiwan	Nested matched case–control study	NHIRD	2000.1.1–2009.12.31	ICD-9-CM	9,304	55,500	≥45 (averag e74.1 ± 8.6)	50.60%	All-cause dementia
Lin et al. (2020)	2020	Taiwan	Retrospective cohort study	NHIRD	2001.1.1–2011.12.31	ICD-9-CM	20,535	82,140	45–80 (average 55.3)	54.10%	All-cause dementia, Alzheimer’s disease, vascular dementia

### 3.3 Bipolar disorder and risk of all-cause dementia

Ten studies (Ma'u et al., 2026; Nielsen et al., 2024; Xu et al., 2024; Liou et al., 2023; Chang et al., 2023; Kershenbaum et al., 2021; Almeida et al., 2016; Chen et al., 2015; Wu et al., 2013; Lin et al., 2020) investigated the association between bipolar disorder (BD) and the risk of all-cause dementia. The pooled analysis using a random-effects model demonstrated that BD was associated with a significantly increased risk of dementia, with extremely high heterogeneity (RR = 3.55; 95% CI = 2.48–5.07, I^2^ = 98.7%, *p* < 0.001) (Figure 2). The leave-one-out sensitivity analysis showed that no individual study substantially altered the pooled risk ratio, indicating that the overall estimate was robust (Supplementary Figure 1A). Additional sensitivity analyses addressing potential population overlap among studies derived from the Taiwan NHIRD showed that the pooled association remained statistically significant regardless of which Taiwanese study was retained, with pooled RRs ranging from 2.40 to 3.08 (all *p* < 0.001). Although the magnitude of the pooled estimates and the degree of heterogeneity varied across these analyses, the direction and statistical significance of the association remained unchanged, supporting the robustness of the main finding. (Additional attachment Table 2) Egger’s regression test revealed no evidence of significant publication bias among the included studies (*p* = 0.479) (Figure 3; Supplementary Figure 2A).

**Figure 2:**
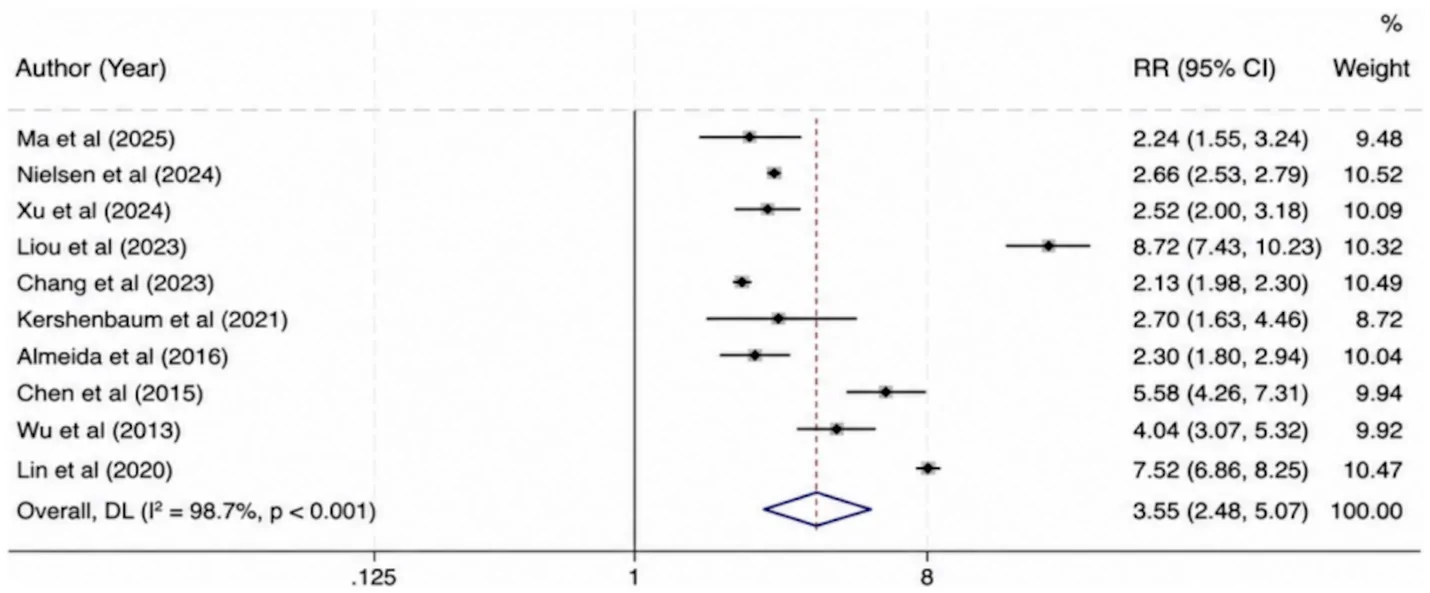
Forest plot of all-cause dementia in any bipolar disorder. Weights are from random-effects model.

**Table 2:** Subgroup analysis of the all-cause dementia in bipolar disorder.

Subgroup	No. study	Test of association			Test of heterogeneity		*p* for subgroup difference
		RR	95%CI	*p* value	*p* value	I^2^	
Follow-up duration(year)							
<10	5	3.89	1.87–8.10	<0.001	<0.001	99.30%	0.613
≥10	5	3.18	2.44–4.14	<0.001	<0.001	89.50%	
Sex							
Male	3	3.18	1.92–5.26	<0.001	0.055	65.60%	0.268
Female	2	4.52	3.14–6.50	<0.001	0.994	0.00%	
Onset age(year)							
<65	2	6.79	2.40–19.21	<0.001	0.006	87.00%	0.680
≥65	2	5.42	4.26–6.89	<0.001	0.14	54.10%	
Cohort study							
Prospective	3	2.38	2.04–2.78	<0.001	0.815	0.00%	0.027
Retrospective	6	4.24	2.61–6.91	<0.001	<0.001	99.20%	

**Figure 3:**
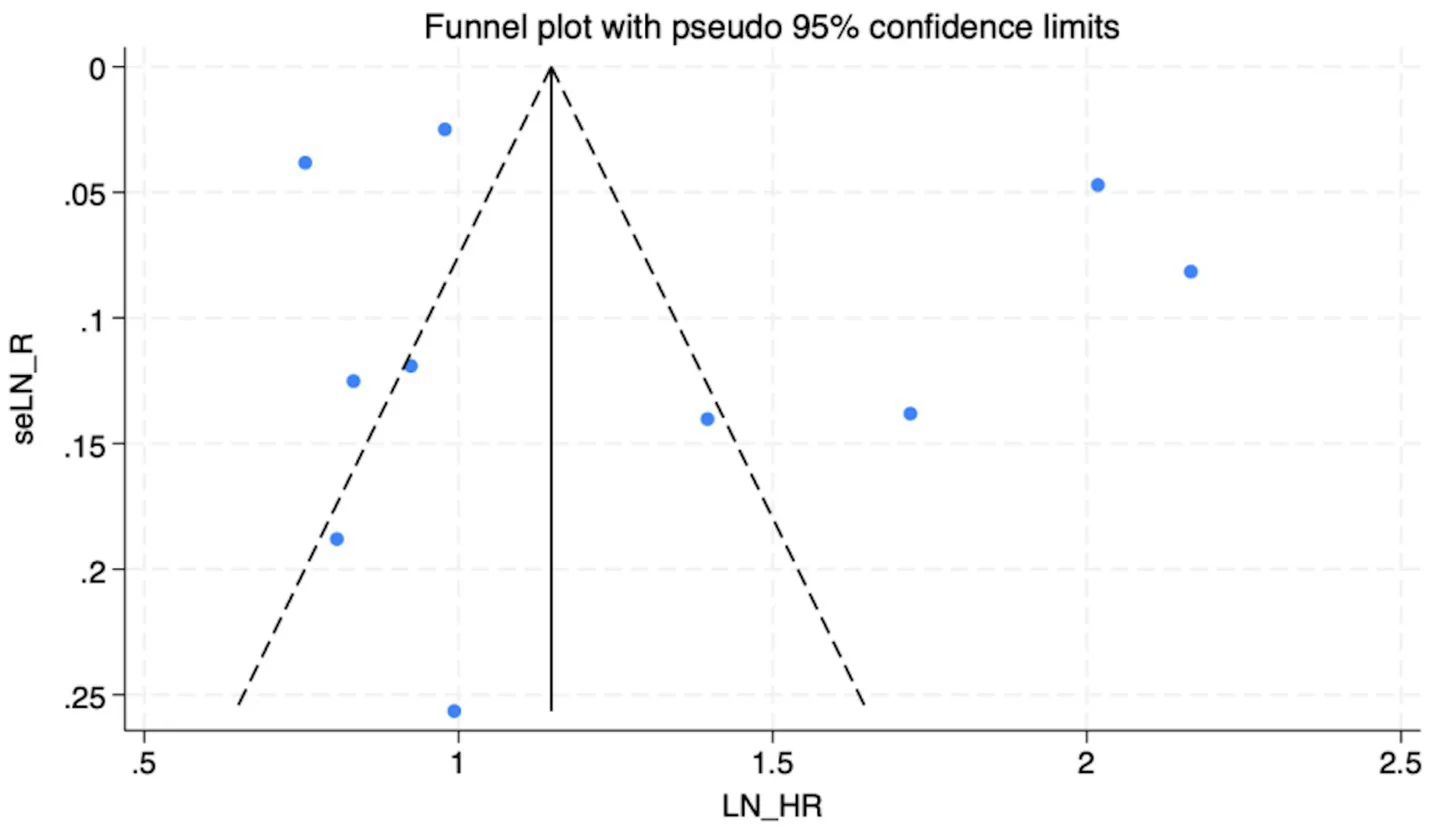
Funnel plot of all-cause dementia.

### 3.4 Bipolar disorder and risk of Alzheimer’s disease

Four studies (Xu et al., 2024; Liu et al., 2024; Liou et al., 2023; Lin et al., 2020) reported the association between bipolar disorder (BD) and the risk of Alzheimer’s disease (AD). The pooled analysis using a random-effects model demonstrated that BD was associated with a significantly increased risk of AD, with extremely high heterogeneity (RR = 5.08; 95% CI: 1.87–13.84, I^2^ = 97.2%, *p* < 0.001) (Figure 4). Sensitivity analysis by sequentially omitting one study at a time showed that although the point estimates of the pooled RR remained above 1 after the exclusion of any single study, the 95% confidence intervals were consistently wide, with lower limits dropping to 0.94 and upper limits reaching 13.84 (Supplementary Figure 1B). This suggests that the statistical significance of the pooled estimate may be sensitive to the exclusion of individual studies; therefore, the robustness of this finding should be interpreted cautiously. Egger’s regression test revealed no evidence of significant publication bias among the included studies (*p* = 0.158) (Figure 5; Supplementary Figure 2B). Nevertheless, given the limited number of included studies (only 4) and the extremely high heterogeneity, this pooled estimate should be interpreted with great caution. Considering that heterogeneity may originate from factors such as region, sex, follow-up duration, and study design, the present study was unable to conduct reliable subgroup analyses owing to the insufficient number of included studies. Future research should incorporate more high-quality cohort studies to perform adequate subgroup analyses and elucidate these potential sources of heterogeneity.

**Figure 4:**
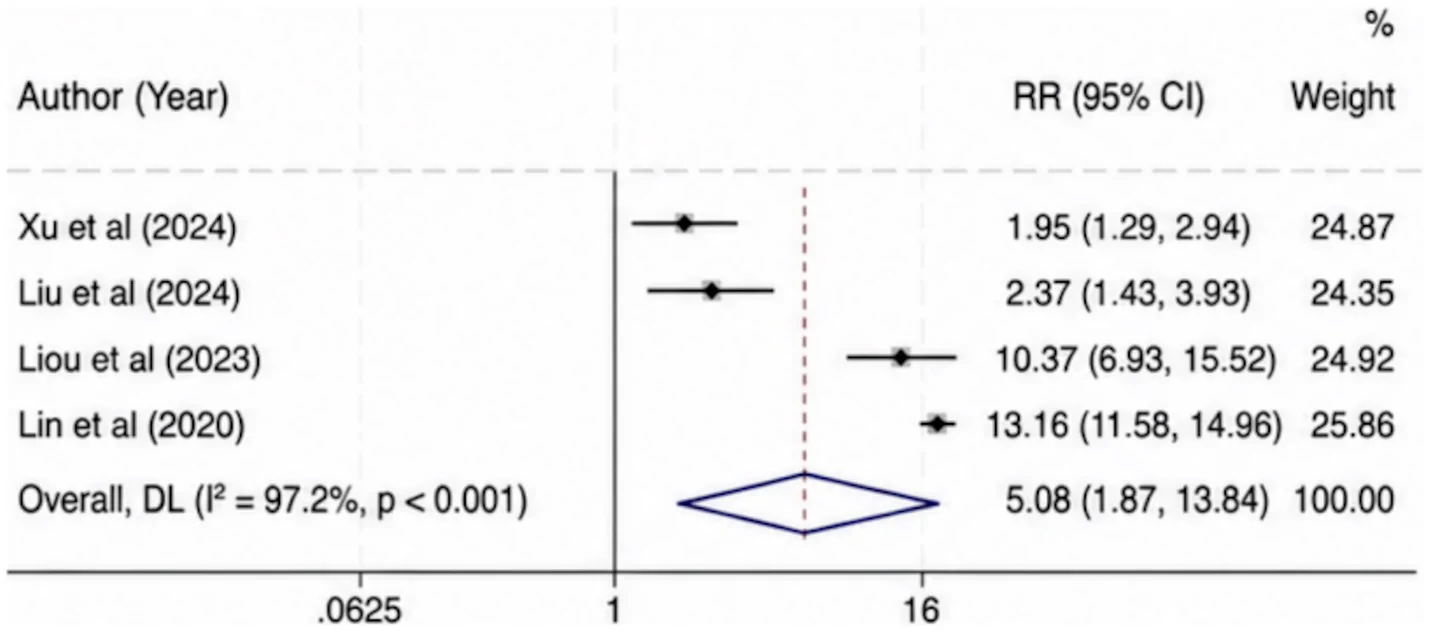
Forest plot of Alzheimer’s disease in any bipolar disorder. Weights are from random-effects model.

**Figure 5:**
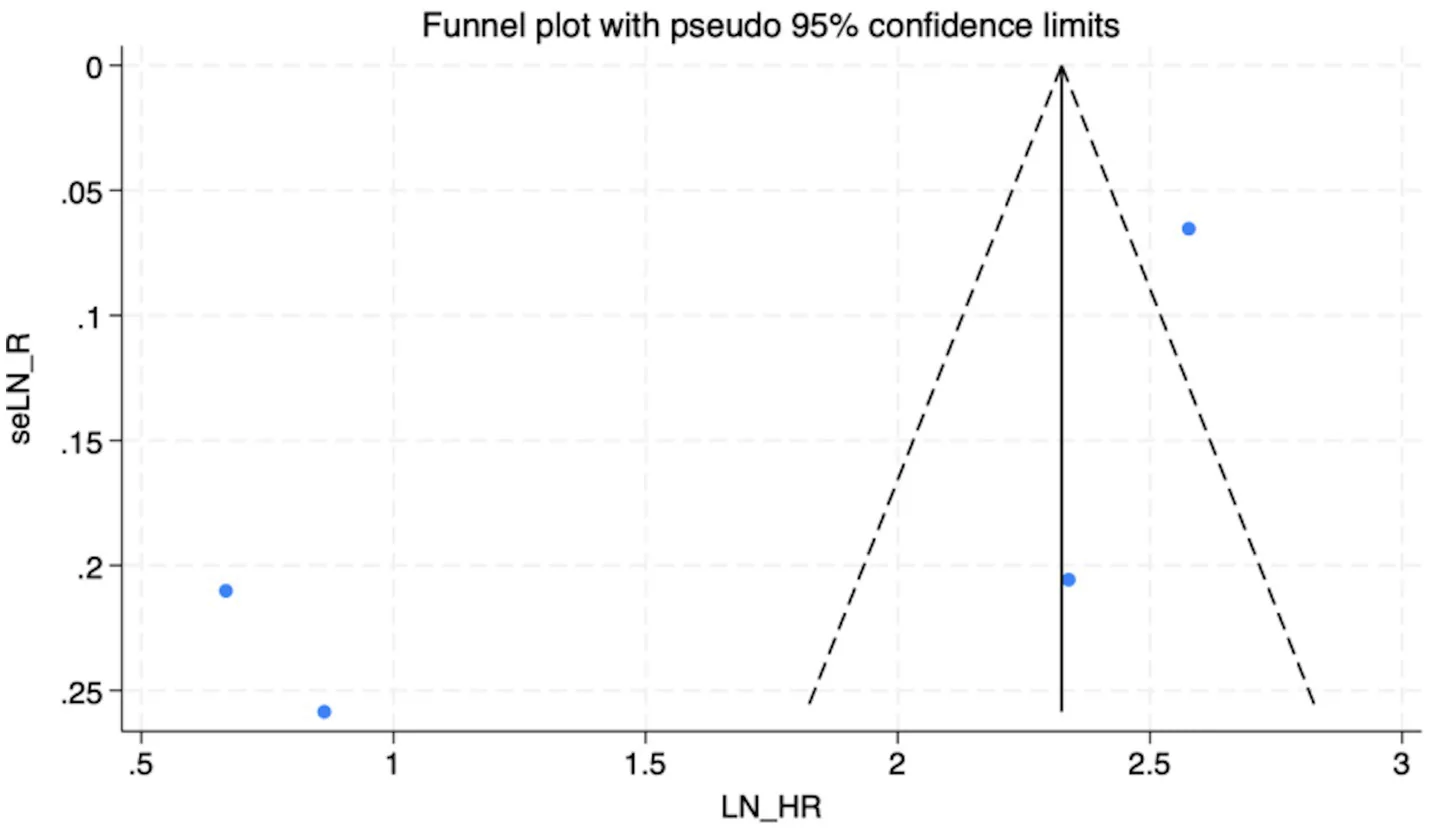
Funnel plot of Alzheimer’s disease.

### 3.5 Bipolar disorder and risk of vascular dementia

Three studies (Xu et al., 2024; Liou et al., 2023; Lin et al., 2020) reported the association between BD and the risk of VaD. The pooled analysis using a random-effects model demonstrated that BD was associated with a significantly increased risk of VaD, with moderate-to-high heterogeneity (RR = 4.33; 95% CI: 3.06–6.13, I^2^ = 71.7%, *p* = 0.029) (Figure 6). Sensitivity analysis by sequentially omitting one study at a time showed that the pooled effect estimate remained statistically significant after the exclusion of each individual study, with no substantial change in the direction of the association, supporting the robustness of the VaD finding (Supplementary Figure 1C). Egger’s regression test revealed no evidence of significant publication bias among the included studies (*p* = 0.204) (Figure 7; Supplementary Figure 2C).

**Figure 6:**
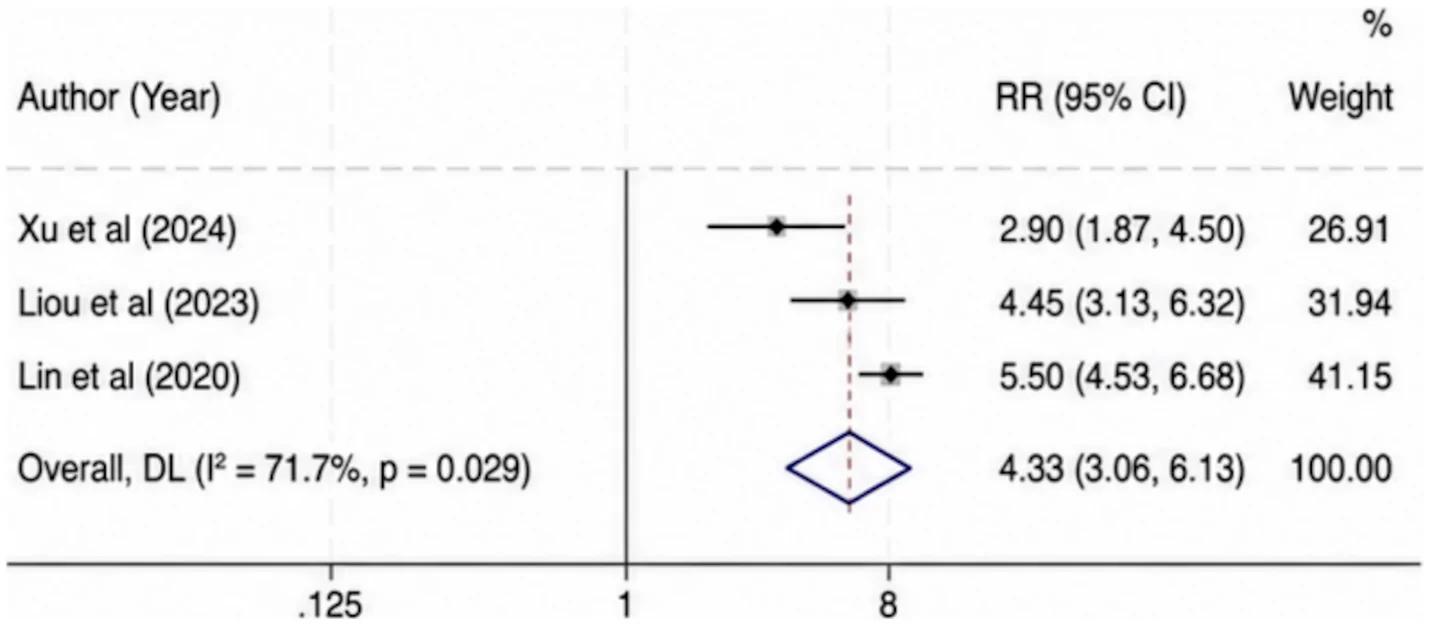
Forest plot of vascular dementia in any bipolar disorder.

**Figure 7:**
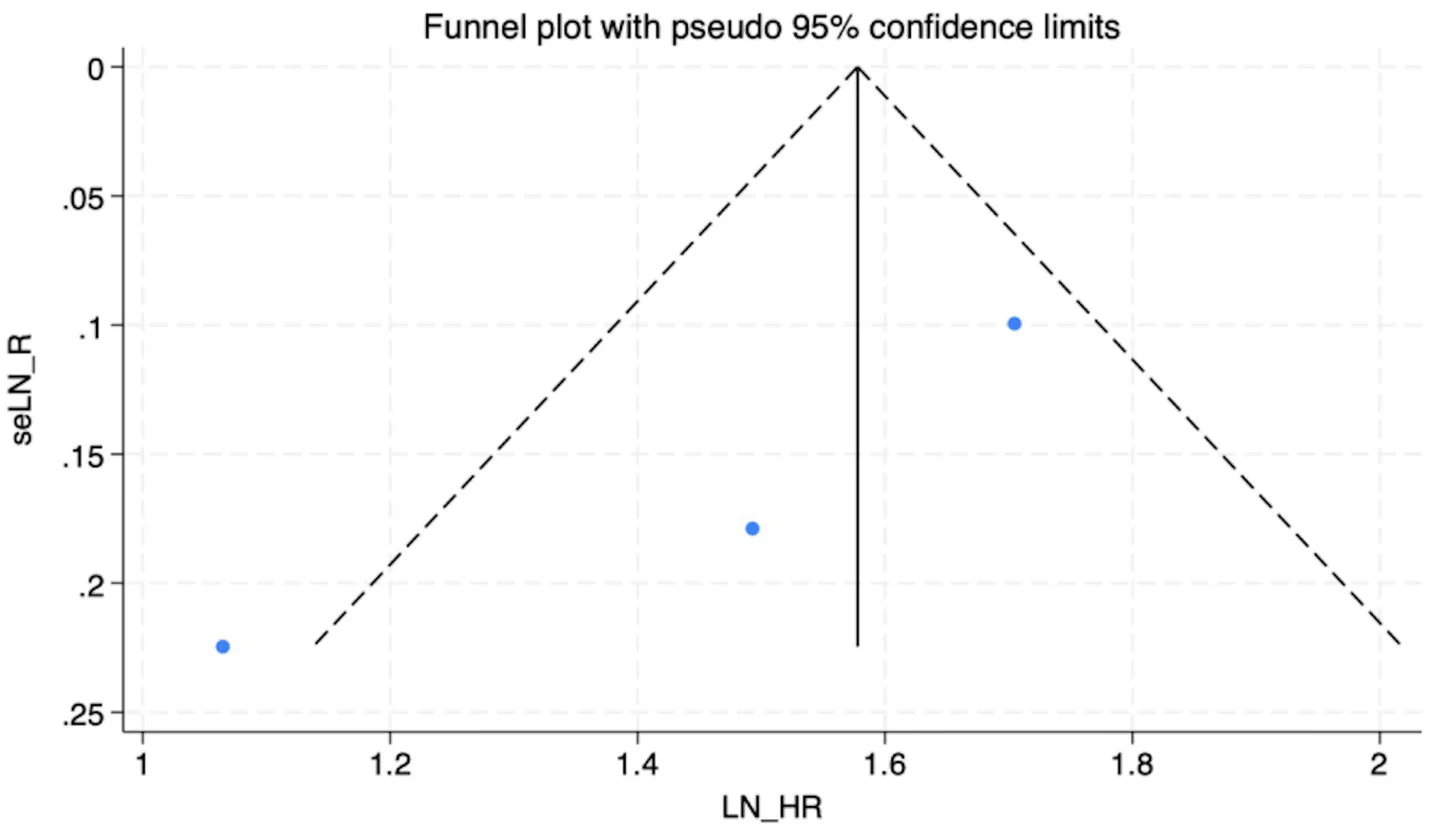
Funnel plot of vascular dementia.

### 3.6 Subgroup analysis

To further explore the potential sources of heterogeneity, we conducted subgroup analyses stratified by follow-up duration, sex, onset age, and study design. Regarding follow-up duration, both studies with <10 years of follow-up (RR = 3.89, 95% CI: 1.87–8.10) and those with ≥10 years (RR = 3.18, 95% CI: 2.44–4.14) demonstrated a significantly increased risk of dementia in patients with BD, although substantial heterogeneity remained in both subgroup (I^2^ = 99.3 and 89.5%, respectively). The test for subgroup differences was not statistically significant (P for interaction = 0.613). By sex,the association was significant in both males (RR = 3.18, 95% CI: 1.92–5.26) and females (RR = 4.52, 95% CI: 3.14–6.50), with no heterogeneity observed in the female subgroup (I^2^ = 0.0%, *p* = 0.994), and moderate heterogeneity in the male subgroup (I^2^ = 65.6%, *p* = 0.055). Although the point estimate was numerically higher in females than in males, the formal test for subgroup differences was not statistically significant (P for interaction = 0.268), providing no evidence of a statistically significant difference in the association between the two sex subgroups. Definitions of early- and late-onset BD varied across the original studies, with different age thresholds used to categorize age at onset. Because individual participant data were unavailable, effect estimates based on different age cutoffs could not be directly reclassified or pooled into uniform onset-age categories. Therefore, the onset-age subgroup analysis was restricted to studies reporting effect estimates that could be classified using a common cutoff of 65 years; studies using other age thresholds were not included in this subgroup analysis. For the purpose of the present subgroup analysis, BD onset before age 65 years was defined as early-onset BD, whereas BD onset at or after age 65 years was defined as late-onset BD. Stratified by onset age, patients with early-onset age (<65 years) (RR = 6.79, 95% CI: 2.40–19.21) and those with late-onset age (≥65 years) (RR = 5.42, 95% CI: 4.26–6.89) both exhibited a significantly elevated risk, with higher heterogeneity in the younger-onset group (I^2^ = 87.0%, *p* = 0.006) and lower heterogeneity in the older-onset group (I^2^ = 54.1%, *p* = 0.145). Although the point estimate was numerically higher among patients with BD onset before age 65 years, the formal test for subgroup differences was not statistically significant (P for interaction = 0.680), indicating no evidence of a statistically significant difference between the two onset-age subgroups. Regarding study design, prospective studies showed a robust association with no heterogeneity (RR = 2.38, 95% CI: 2.04–2.78, I^2^ = 0.0%, *p* = 0.815), whereas retrospective studies yielded a higher effect estimate but with extremely high heterogeneity (RR = 4.24, 95% CI: 2.61–6.91, I^2^ = 99.2%) (Table 2). The test for subgroup differences by study design was statistically significant (P for interaction = 0.027), indicating a statistically significant difference in the pooled effect estimates between prospective and retrospective studies. Overall, the association between BD and increased dementia risk was observed across the examined subgroups. Although higher point estimates were observed in females and in patients with BD onset before age 65 years, the corresponding tests for subgroup differences were not statistically significant. Therefore, these numerical differences should not be interpreted as evidence of statistically significant effect modification by sex or onset age.

## 4 Discussion

### 4.1 Main finding

This systematic review and meta-analysis shows consistent associations between bipolar disorder (BD) and elevated dementia risk, covering all-cause dementia as well as distinct dementia subtypes. Pooled data from included longitudinal observational studies demonstrate substantially higher all-cause dementia risk among people living with BD. This association remained stable across sensitivity analyses, with no evidence of meaningful publication bias. These results identify BD as an important high-risk condition for subsequent cognitive decline and incident dementia. Beyond all-cause dementia, BD was associated with raised risks of both AD and VaD. The AD-related finding requires cautious interpretation given the small number of primary studies and its sensitivity to individual-study influence. By contrast, the BD-VaD association was more robust. Consistent risk elevation across dementia phenotypes underscores the broad impact of BD on diverse pathological forms of cognitive impairment. Subgroup analyses showed excess dementia risk persisted across varying follow-up durations, sex groups, illness onset ages, and cohort study designs. Although the pooled effect estimates were numerically higher in females and in patients with BD onset before age 65 years, formal tests for subgroup differences were not statistically significant for sex or age at BD onset. Therefore, these numerical differences should not be interpreted as evidence of effect modification. In contrast, the test for subgroup differences by study design was statistically significant, with retrospective studies yielding a higher pooled effect estimate and substantially greater heterogeneity than prospective studies. However, this finding should be interpreted cautiously given the limited number of studies within the subgroups. The included studies were of moderate-to-high methodological quality according to the NOS, and all applied multivariable adjustment for potential confounders. Nevertheless, substantial between-study heterogeneity and residual confounding should be considered when interpreting the pooled estimates.

### 4.2 Comparison with previous studies

Our results align broadly with population-based cohort research conducted in New Zealand, Denmark, the United Kingdom, and Taiwan, most of which report at least a two-fold dementia risk elevation for individuals with BD. Drawing on cross-regional data, this meta-analysis synthesised more than 1.53 million participants and over 90,000 dementia cases. This large sample improves statistical power relative to single-cohort investigations and generates more precise summary risk estimates. Pooling also attenuated the weight of extreme effect sizes reported in some individual studies such as risk ratios above 7 (Liou et al., 2023; Lin et al., 2020). Subgroup and sensitivity analyses indicate heterogeneity across earlier cohorts may stem from study design, population characteristics, and follow-up length. Unlike most prior primary work focused solely on all-cause dementia, our analysis synthesised AD and VaD separately and provides quantitative evidence linking BD to increased risk for both major dementia subtypes.

Relative to earlier meta-analyses, the present study offers several key advances. Past quantitative syntheses mainly examined overall BD-related all-cause dementia risk. Diniz et al. (2017) published the first meta-analysis on this topic with only six included studies and no systematic evaluation of dementia subtypes. A subsequent meta-analysis by Kessing and Munkholm (2026) expanded the study pool yet noted persistent between-study heterogeneity without comprehensive assessment of subtype-specific risks. Our work incorporates recently published large-scale cohort data from Europe, Oceania, and Taiwan to deliver geographically broader and updated evidence. While earlier meta-analyses largely focused on pooled summary effects with limited exploration of heterogeneity sources, we performed pre-specified subgroup analyses stratified by follow-up duration, sex, age at BD onset, and study design. This enables more systematic evaluation of factors driving cross-study variability. Critically, we analysed AD and VaD rather than restricting outcomes to all-cause dementia. Even so, few studies report subtype-specific endpoints, and sensitivity testing confirms instability for AD-related estimates, which reinforces the need for cautious interpretation of subtype-specific findings.

### 4.3 Interpretation of findings

Multiple biological pathways may underlie the higher dementia risk observed among people with BD. However, the biological mechanisms underlying this association should be interpreted cautiously. Cognitive impairment associated with BD, clinical or neuroprogressive changes during the course of the illness, and the subsequent development of neurodegenerative dementia are related but conceptually distinct constructs. Persistent cognitive impairment may be present even during euthymic phases of BD, whereas neurodegenerative dementia represents a clinically distinct condition typically characterized by progressive decline that interferes with independent functioning. Although these processes may coexist and potentially share certain biological pathways, cognitive impairment in BD should not be equated with neurodegenerative dementia or assumed to inevitably progress to dementia. First, chronic neuroinflammation represents a plausible mechanism linking recurrent mood episodes to progressive cognitive deterioration. BD features immune dysregulation and persistent low-grade inflammation, and inflammatory biomarkers correlate with cognitive impairment. Neuroinflammatory changes including microglial dysfunction can impair synaptic plasticity, increase oxidative stress, and disrupt neuronal function within cognition-relevant brain regions such as the hippocampus and prefrontal cortex (Rosenblat et al., 2015; Rolstad et al., 2015). These mechanisms may contribute to cognitive vulnerability in BD and may overlap with biological processes implicated in AD-related neurodegeneration; however, such overlap does not establish a direct neuroprogressive pathway from BD to AD. Second, vascular damage may explain elevated VaD risk. Individuals with BD carry heavier burdens of cardiovascular and metabolic comorbidities including hypertension, metabolic disturbances, and adverse lifestyle exposures (Goldstein et al., 2015). Together with physiological stress during manic episodes, these vascular risk factors may contribute to cerebrovascular injury and small-vessel disease and increase susceptibility to vascular cognitive impairment. This proposed mechanism is congruent with our significant BD-VaD association. Third, hypothalamic–pituitary–adrenal (HPA) axis dysregulation may contribute to cognitive decline in BD. Existing evidence links BD to disrupted HPA-axis function and altered cortisol, which can compromise hippocampal integrity, neuroplasticity, and stress-related neuronal function (Belvederi Murri et al., 2016). Greater illness severity and a higher cumulative burden of manic and depressive episodes may be accompanied by prolonged exposure to inflammatory, vascular, neuroendocrine, and psychosocial stressors, potentially contributing to cognitive vulnerability over the course of BD. Recent staging frameworks provide an additional perspective on the longitudinal course of BD. Clinical staging models conceptualize BD as a heterogeneous illness in which increasing illness burden may be accompanied by changes in recurrence, functioning, cognition, and neurobiological characteristics across different stages. Dell’Osso et al. highlighted the potential value of integrating clinical manifestations with neurobiological markers in multidimensional staging models, while also noting that currently proposed staging systems remain heterogeneous and have limited routine clinical application (Dell'Osso et al., 2024). More recently, Caporali et al. (2026) identified stage-related differences in functional brain network architecture, providing neuroimaging evidence that different clinical stages of BD may be associated with distinct neurobiological characteristics. These findings provide a useful framework for understanding how cumulative illness burden may be accompanied by clinical and neurobiological changes over the course of BD. However, BD staging and neuroprogression should not be interpreted as equivalent to neurodegenerative dementia, and current evidence does not establish that progression through BD stages represents a direct pathway to subsequent dementia. Persistent cognitive deficits have also been documented during euthymic phases of BD (Cullen et al., 2016; Alonso-Lana et al., 2016), supporting the possibility that cognitive impairment is not confined to acute mood episodes. Nevertheless, the presence or accumulation of such cognitive deficits should not be interpreted as evidence that BD-related cognitive impairment necessarily progresses to neurodegenerative dementia. However, although such cumulative mechanisms are biologically plausible, our subgroup analysis did not provide evidence that dementia risk differed significantly according to onset-age category. Finally, clinical and behavioural factors can further heighten cognitive vulnerability. Long-term medication exposure, limited social contact, social isolation, poor treatment adherence, and unhealthy behaviours may interact with inflammatory, vascular, and neuroendocrine disturbances to influence cognitive. Pharmacological exposure may also influence subsequent dementia risk, particularly lithium treatment, which has been highlighted as a potentially relevant modifier in previous evidence (Velosa et al., 2020). Disentangling these extrinsic contributors from illness-intrinsic mechanisms remains challenging, yet they constitute plausible additional pathways toward dementia among patients with BD. Alternative explanations for the observed association should also be considered. Reverse causation cannot be completely excluded, particularly in individuals whose first diagnosis of BD occurs later in life. Affective or behavioural symptoms may, in some individuals, occur during the prodromal phase of an as-yet-unrecognized neurodegenerative disorder and precede the eventual diagnosis of dementia. Thus, at least part of the observed association between BD and subsequent dementia in observational studies could reflect preclinical neurodegenerative changes rather than a unidirectional causal pathway from BD to dementia. In addition, vascular and metabolic comorbidities, illness severity, cumulative episode burden, and treatment exposure may act as confounding or intermediate factors and may partly account for variation in dementia risk across studies. Because the available evidence is observational, the present meta-analysis cannot determine the relative contributions of these mechanisms or establish a causal neuroprogressive pathway from BD to dementia.

Subgroup analyses showed numerical variation in the magnitude of the association, but these differences should be interpreted cautiously. Although the pooled RR was numerically higher in women than in men, the formal test for subgroup differences was not statistically significant (P for subgroup difference = 0.268). Therefore, the present findings do not provide statistical evidence that sex modifies the association between BD and dementia risk. Sex-related differences in illness trajectory, reproductive hormonal changes, psychiatric characteristics, inflammatory processes, and vascular risk burden remain biologically plausible factors that warrant further investigation (Leibenluft, 1996); however, the current subgroup findings do not establish a significantly greater dementia risk among women with BD. Similarly, the pooled RR was numerically higher in the early-onset BD subgroup (<65 years) than in the late-onset BD subgroup (≥65 years), but the difference between the two subgroups was not statistically significant (P for subgroup difference = 0.680). Definitions of early- and late-onset BD varied across the original studies, with different age thresholds used to categorize age at onset. Because individual participant data were unavailable, effect estimates based on different age thresholds could not be directly reclassified or pooled into uniform onset-age categories. Therefore, the onset-age subgroup analysis was restricted to studies reporting effect estimates that could be classified using a common cutoff of 65 years; studies using other age thresholds were not included in this subgroup analysis. For the purpose of the present subgroup analysis, BD onset before age 65 years was defined as early-onset BD, whereas BD onset at or after age 65 years was defined as late-onset BD. Consequently, the numerically higher point estimate observed in the early-onset subgroup should not be interpreted as evidence of a statistically significantly greater dementia risk in early-onset BD, and the inconsistency in onset-age definitions across studies should be considered when interpreting these findings. Higher point estimates and marked heterogeneity within retrospective cohorts may partly stem from ascertainment and detection bias. In particular, patients with BD generally have more frequent contact with healthcare services and greater opportunities for clinical follow-up than individuals without BD, increasing the likelihood that cognitive symptoms are assessed and dementia is detected and recorded. This differential surveillance could contribute to an overestimation of the observed association between BD and dementia, particularly in retrospective register-based studies. By comparison, prospective cohorts implement more standardised follow-up and outcome verification. The formal test for subgroup differences by study design was statistically significant (P for subgroup difference = 0.027), indicating a statistically significant difference in the pooled effect estimates between prospective and retrospective studies. Nevertheless, this finding should be interpreted cautiously given the limited number of studies and the substantial heterogeneity among retrospective studies. The association between BD and dementia risk was observed in both the shorter- and longer-follow-up subgroups; however, the formal test for subgroup differences was not statistically significant (P for subgroup difference = 0.613). Therefore, the current findings do not provide evidence that the magnitude of the association differs significantly according to follow-up duration. Although cognitive impairment has been reported in relatively young and euthymic patients with BD, this observation should not be interpreted as evidence that such impairment necessarily represents an early stage of, or inevitably progresses to, neurodegenerative dementia. Further longitudinal studies with detailed information on illness course, episode burden, vascular comorbidities, treatment exposure, and standardized dementia ascertainment are needed to clarify the temporal and potentially causal relationships between BD-related cognitive impairment and subsequent dementia.

### 4.4 Limitations

This study has several limitations. First, the geographic coverage of the included evidence was limited, with studies conducted mainly in Oceania, Europe, and Taiwan, China; therefore, the generalizability of our findings to mainland China, other Asian countries, Africa, and the Americas remains uncertain. Second, the evidence for dementia subtypes was limited, with only four studies evaluating AD and three evaluating VaD, which prevented reliable multidimensional subgroup analyses; moreover, the sensitivity analysis indicated that the statistical significance of the AD association might be lost after exclusion of individual studies, warranting cautious interpretation. In addition, none of the included studies separately reported outcome data for Lewy body dementia (LBD) or frontotemporal dementia (FTD), precluding quantitative synthesis of these two dementia subtypes. Future cohort studies should therefore report a broader range of subtype-specific dementia outcomes, including LBD and FTD in addition to AD and VaD, to enable more comprehensive assessments of the association between BD and specific dementia subtypes. Third, substantial heterogeneity could not be completely resolved. Formal tests for subgroup differences suggested that study design may contribute to between-study heterogeneity, whereas no statistically significant subgroup differences were detected by sex or age at onset. However, the small number of studies within several subgroups may have limited the statistical power to detect between-subgroup differences. The available data did not permit further stratification or adjustment for potentially important factors such as medication type, illness duration, education, and physical comorbidities. In addition, the definitions of early- and late-onset BD were not consistent across the included studies, with different age thresholds used to categorize age at onset. Because individual participant data were unavailable, effect estimates based on different thresholds could not be reclassified using a uniform definition. Consequently, the onset-age subgroup analysis was restricted to studies that allowed classification using the common cutoff of 65 years (<65 vs. ≥65 years), which reduced the number of studies available for this subgroup analysis and may have limited the statistical power and precision of the between-subgroup comparison. Therefore, the findings regarding age at onset should be interpreted with caution. In addition, several included studies were derived from the Taiwan National Health Insurance Research Database (NHIRD), with partially overlapping observation periods, raising the possibility of overlapping study populations and potential double-counting of participants. To assess the potential influence of this issue, we conducted additional sensitivity analyses by retaining only one of these Taiwanese studies at a time while excluding the remaining potentially overlapping studies. Although the direction and statistical significance of the pooled association remained unchanged across these analyses, potential population overlap cannot be completely excluded and therefore remains a possible source of bias in the present meta-analysis. Fourth, dementia ascertainment in the included studies was based on ICD-9 or ICD-10 diagnostic codes, primarily derived from administrative or registry-based data rather than standardized, study-specific cognitive assessments. Therefore, incomplete case ascertainment or diagnostic misclassification cannot be entirely excluded (McGuinness et al., 2019; Wilkinson et al., 2018). The limited representation of sex-specific populations is another concern, as only one included study consisted exclusively of men. Fifth, because all included evidence was observational, our findings demonstrate associations rather than causality, and residual confounding and reverse causation cannot be completely excluded (Ho, 2025). Finally, our search was restricted to four bibliographic databases and published English-language studies, and conference abstracts and other grey literature were excluded. In addition, the number of studies included in our meta-analysis differed from that in some previous meta-analyses, partly because of differences in eligibility criteria, including the definitions of eligible exposures, comparators, outcomes, study designs, and the availability of effect estimates suitable for quantitative synthesis. Therefore, although we updated the literature search and carefully reassessed potentially eligible studies, differences in study-selection criteria across meta-analyses may have resulted in differences in the evidence base included. Moreover, although Egger’s tests did not indicate significant publication bias, the possibility of bias from unpublished or selectively reported evidence cannot be completely ruled out (McKenzie et al., 2024).

In addition, none of the included studies provided effect estimates adjusted for lithium treatment or stratified by lithium exposure. Previous evidence has suggested that lithium may have potential neuroprotective effects against Alzheimer’s disease (Velosa et al., 2020); therefore, the inability to account for lithium exposure may represent a potential contributor to the substantial heterogeneity and instability observed in the pooled AD results. Quantitatively evaluating the potential modifying effect of lithium treatment on dementia risk would require individual participant data (IPD) meta-analyses or future primary studies providing sufficiently detailed lithium-adjusted or lithium-stratified estimates.

### 4.5 Implication

Our findings carry practical implications for clinical practice. Within psychiatric services, routine follow-up for patients with BD should incorporate cognitive screening to address long-term cognitive outcomes. The International Society for Bipolar Disorders recommends formal cognitive assessment for partially or fully remitted patients and endorses brief, feasible screening instruments for real-world clinical use (Miskowiak et al., 2018). Clinical management should also target modifiable vascular and metabolic risk factors including blood-pressure control, glycaemic status, sleep health, and physical activity. Within geriatric memory clinics, clinicians should actively enquire about past BD history when evaluating new-onset cognitive impairment or dementia. Psychiatric and neurological assessment should be coordinated as appropriate. Patients with high cumulative risk burden may benefit from intensified preventive measures such as regular physical exercise and sustained social engagement consistent with established dementia-prevention guidance (Cullen et al., 2016; Livingston et al., 2024). Future research should include prospective cohorts applying uniform cognitive assessment tools and broader geographic sampling including mainland China and other under-represented populations. Larger datasets for AD and VaD outcomes are required to generate stable subtype-specific risk estimates and clarify whether mood pharmacotherapies modify subsequent dementia risk. Importantly, future primary cohort studies should systematically report exposure to lithium and other mood stabilizers and, where possible, provide dementia outcomes stratified by treatment exposure or effect estimates adjusted for these treatments. Such data would help clarify whether pharmacological treatment modifies the subsequent risk of dementia among individuals with BD. Mechanistic work should further investigate inflammatory and vascular pathways linking BD to neurodegeneration. Causal-inference methods such as Mendelian randomisation may help separate true causal effects from residual confounding. Given that many dementia risk factors are modifiable, individuals with BD may warrant greater attention within dementia-prevention frameworks. Integrated long-term surveillance combining psychiatric and cognitive outcomes, together with public-health education and management of modifiable risk factors, may help support dementia-prevention efforts in this population (Diniz et al., 2017; Livingston et al., 2024).

## 5 Conclusion

This meta-analysis shows that BD is associated with an increased risk of all-cause dementia, Alzheimer’s disease, and vascular dementia. Although the pooled effect estimates varied across subgroups, formal tests showed no statistically significant subgroup differences according to sex, age at BD onset, or follow-up duration, whereas a significant subgroup difference was observed by study design. Given the substantial between-study heterogeneity, limited subtype-specific data, restricted geographical scope, and the observational nature of the included studies, causal inference cannot be drawn and the findings should be interpreted cautiously. Further high-quality longitudinal studies are needed to clarify the sources of heterogeneity and the factors underlying the association between BD and dementia.

## Data Availability

The original contributions presented in the study are included in the article/Supplementary material, further inquiries can be directed to the corresponding author.
