# Supplementary material for: Bipolar disorder and risk of all-cause and specific dementia: a systematic review and meta-analysis
Source: Front Aging Neurosci. 2026 Oct 13;18:1980508. doi: 10.3389/fnagi.2026.1980508 (PMC13710222; doi:10.3389/fnagi.2026.1980508)
Supplement: Supplementary file 1 [file Data_Sheet_1.docx]

**Supplementary Tables 1 Search strategy of Pubmed**

| No. | Content | Result |
| --- | --- | --- |
| #1 | Search: "Bipolar Disorder"[Mesh] Sort by: Most Recent | 48,649 |
| #2 | Search: ((((((Bipolar Disorder*[Title/Abstract]) OR (Bipolar Mood Disorder*[Title/Abstract])) OR (Manic Depression*[Title/Abstract])) OR (Bipolar Affective Psychos*[Title/Abstract])) OR (Manic Depressive Psychos*[Title/Abstract])) OR (Bipolar Depression*[Title/Abstract])) OR (Manic Disorder*[Title/Abstract]) Sort by: Most Recent | 45,260 |
| #3 | Search: (#1) OR (#2) Sort by: Most Recent | 65,962 |
| #4 | Search: "Dementia"[Mesh] Sort by: Most Recent | 238,457 |
| #5 | Search: (Dementia*[Title/Abstract]) OR (Amentia*[Title/Abstract])Sort by: Most Recent | 177,168 |
| #6 | Search: (#4) OR (#5) Sort by: Most Recent | 310,275 |
| #7 | Search: "Risk"[Mesh] Sort by: Most Recent | 1,531,363 |
| #8 | Search: Risk*[Title/Abstract] Sort by: Most Recent | 3,653,454 |
| #9 | Search: (#7) OR (#8) Sort by: Most Recent | 4,164,210 |
| #10 | Search: ((#3) AND (#6)) AND (#9) Sort by: Most Recent | 432 |

**Supplementary Tables 2 Search strategy of EMBASE**

| No. | Content | Result |
| --- | --- | --- |
| #1 | 'bipolar disorder'/exp | 111,654 |
| #2 | 'bipolar disorder*':ab,ti OR 'bipolar mood disorder*':ab,ti OR 'manic depression*':ab,ti OR 'bipolar affective psychos*':ab,ti OR 'manic depressive psychos*':ab,ti OR 'bipolar depression*':ab,ti OR 'manic disorder*':ab,ti | 69,783 |
| #3 | #1 OR #2 | 118,588 |
| #4 | 'dementia'/exp | 574,028 |
| #5 | dementia*:ab,ti OR amentia*:ab,ti | 258,607 |
| #6 | #4 OR #5 | 610,777 |
| #7 | 'risk'/exp | 3,732,112 |
| #8 | risk*:ab,ti | 5,358,974 |
| #9 | #7 OR #8 | 6,343,130 |
| #10 | #3 AND #6 AND #9 | 2,146 |

**Supplementary Tables 3 Search strategy of Cochrane Library**

| No. | Content | Result |
| --- | --- | --- |
| #1 | MeSH descriptor: [Bipolar Disorder] this term only | 3,716 |
| #2 | (Bipolar Disorder*):ti,ab,kw OR (Bipolar Mood Disorder*):ti,ab,kw OR (Manic Depression*):ti,ab,kw OR (Bipolar Affective Psychos*):ti,ab,kw OR (Manic Depressive Psychos*):ti,ab,kw | 8,966 |
| #3 | (Bipolar Depression*):ti,ab,kw OR (Manic Disorder*):ti,ab,kw | 5,792 |
| #4 | #1 OR #2 OR #3 | 9,346 |
| #5 | MeSH descriptor: [Dementia] this term only | 4,548 |
| #6 | (Dementia*):ti,ab,kw OR (Amentia*):ti,ab,kw | 20,887 |
| #7 | #5 OR #6 | 20,887 |
| #8 | MeSH descriptor: [Risk] this term only | 4,528 |
| #9 | (Risk*):ti,ab,kw | 351,634 |
| #10 | #8 OR #9 | 351,634 |
| #11 | #4 AND #7 AND #10 | 54 |

**Supplementary Tables 4 Search strategy of Web of science**

| No. | Content | Result |
| --- | --- | --- |
| #1 | (((((((TS=(Bipolar Disorder)) OR TS=(Bipolar Disorder*)) OR TS=(Bipolar Mood Disorder*)) OR TS=(Manic Depression*)) OR TS=(Bipolar Affective Psychos*)) OR TS=(Manic Depressive Psychos*)) OR TS=(Bipolar Depression*)) OR TS=(Manic Disorder*) | 85,097 |
| #2 | ((TS=(Dementia)) OR TS=(Dementia*)) OR TS=(Amentia*) | 256,800 |
| #3 | (TS=(Risk)) OR TS=(Risk*) | 5,284,155 |
| #4 | #1 AND #2 AND #3 | 649 |

**Supplementary Tables 5 The quality assessment of cohort studies**

| Study | Year | Selection | Comparability | Outcome | Total |
| --- | --- | --- | --- | --- | --- |
| Cohort studies (n=10) | | | | | |
| Etuini Ma’u | 2025 | **** | ** | ** | 8 |
| Jane Lykke Nielsen | 2024 | **** | ** | ** | 8 |
| Xinming Xu | 2024 | *** | ** | ** | 7 |
| Yiliang Liu | 2024 | *** | ** | ** | 7 |
| Ying‑Jay Liou | 2023 | **** | ** | ** | 8 |
| Wei Hung Chang | 2023 | **** | ** | ** | 8 |
| Anne Kershenbaum | 2021 | ** | * | ** | 5 |
| Osvaldo P. Almeida | 2016 | **** | * | *** | 8 |
| Mu-Hong Chen MD | 2015 | **** | * | ** | 7 |
| Shen-Hsiung Lin M.D. | 2020 | **** | ** | ** | 8 |
| Case-control studies (n=1) | | | | | |
| Wu K-Y | 2013 | **** | ** | ** | 8 |

**Supplementary Figure 1.A** Sensitivity analysis of all-cause dementia

**Supplementary Figure 1.B** Sensitivity analysis of Altheimer’s disease

**Supplementary Figure 1.C** Sensitivity analysis of vascular dementia

**Supplementary Figure 2.A** Publication bias test of all-cause dementia

**Supplementary Figure 2.B** Publication bias test of Altheimer’s disease

**Supplementary Figure 2.C** Publication bias test of vascular dementia
